# Vector Competence for West Nile Virus Lineage 1 and 2: Temperature Dependency in Temperate *Culex pipiens* Mosquitoes

**DOI:** 10.3390/microorganisms14092084

**Published:** 2026-09-17

**Authors:** Estela Gonzalez, Insiyah Parekh, Sanam Thakkar, Henry F. Ashpitel, Mohammed Salman, Stuart Ackroyd, Alejandro Nunez, Audra-Lynn Schlachter, Nicholas Johnson, Karen L. Mansfield

**Affiliations:** 1 Vector-Borne Diseases Group, Virology Department, Animal and Plant Health Agency, Woodham Lane, New Haw, Addlestone KT15 3NB, UK; insiyah.parekh@apha.gov.uk (I.P.); sanam.thakkar@apha.gov.uk (S.T.); nick.johnson@apha.gov.uk (N.J.); karen.mansfield@apha.gov.uk (K.L.M.); 2 School of Life Sciences, University of Sussex, Brighton BN1 9QG, UK; 3 Pathology & Animal Sciences Department, Animal and Plant Health Agency, Woodham Lane, New Haw, Addlestone KT15 3NB, UK; henry.ashpitel@apha.gov.uk (H.F.A.); mohammed.salman@apha.gov.uk (M.S.); stuart.ackroyd@apha.gov.uk (S.A.); alejandro.nunez@apha.gov.uk (A.N.); audra-lynne.schlachter@apha.gov.uk (A.-L.S.); 4 Faculty of Health and Medical Sciences, University of Surrey, Guildford GU2 7XH, UK

**Keywords:** vector-borne disease, *Culex*, climate change, arbovirus

## Abstract

West Nile virus (WNV) is a mosquito-borne pathogen predominantly transmitted by *Culex* mosquitoes. Although birds are the primary hosts, other vertebrates, such as humans and horses, can also be affected. WNV incidence is increasing globally, expanding to northerly latitudes. This study investigated the vector competence of UK *Culex pipiens* exposed to WNV lineage 1 (Lin1) and lineage 2 (Lin2) at temperatures frequently experienced during northern European summers. Colonised *Cx. pipiens* exposed to blood meals containing WNV Lin1 and Lin2 were maintained at 20 °C or 25 °C and sampled at 7, 14, and 21 days post-infection (DPI) to assess infection, dissemination, and transmission potential. Differences between lineages were observed; at 20 °C, dissemination and transmission potential were greater for Lin1, whereas at 25 °C both lineages disseminated and showed transmission potential, with consistently higher rates for Lin2. These findings indicate that *Cx. pipiens* demonstrate transmission potential for both WNV Lin1 and Lin2 at temperatures representative of current northern European summers, with Lin2 showing greater transmission efficiency, particularly at 25 °C, while Lin1 was less efficient but remained active across a broader temperature range. These data highlight the potential risk of this virus in temperate areas currently free of this mosquito-borne virus.

## 1. Introduction

Mosquitoes are among the most important vectors of infectious diseases worldwide, transmitting pathogens that affect both human and animal health. These infections range from mild febrile illnesses to severe, life-threatening conditions, and their capacity to cause outbreaks emphasises their public health significance [1]. Traditionally, mosquito-borne diseases (MBDs) were considered a major concern in tropical and subtropical regions; however, their epidemiology has shifted significantly in recent decades mainly due to climate change, globalisation, and anthropogenic environmental modifications. These factors have collectively facilitated the expansion of mosquito species and the pathogens they carry, resulting in a risk of potential transmission and an increasing incidence of MBDs in temperate zones, including Europe [2]. Europe largely eliminated mosquito-transmitted pathogens by the mid-20th century through vector control and improved living conditions [3]. However, the continent is now witnessing a resurgence of these diseases driven by multiple factors: rising temperatures, prolonged summers, extreme weather events, such as floods, becoming more frequent, and milder winters that have extended the seasonal activity of native mosquito species such as *Culex pipiens*, a primary vector of West Nile virus (WNV) and Usutu virus (USUV) in Europe. These climatic changes can also favour mosquito overwintering and survival, and accelerate viral replication within vectors, increasing transmission potential [4]. Additionally, globalisation and environmental changes have facilitated the introduction, establishment and further expansion of the invasive mosquito species *Aedes albopictus* (Asian tiger mosquito), *Ae. japonicus*, *Ae. koreicus*, and the incursion of *Ae. aegypti* (yellow fever mosquito), which are competent vectors for arboviruses such as dengue virus, chikungunya virus, and Zika virus. As a result, the epidemiological consequences of these shifts are evident in the rising number of locally acquired cases of arboviral infections across Europe. West Nile virus, once sporadic, now causes recurrent outbreaks in southern and central Europe, with lineage 1 (Lin1) and lineage 2 (Lin2) spreading through the continent and with cases reported as far north as Germany, Belgium, and the Netherlands [5,6,7]. In 2025, and as of 3 December 2025, over 1100 locally acquired WNV infections were documented across 14 European countries, accompanied by significant mortality [8,9]. Moreover, a potential incursion of WNV in 2023 has been found in a retrospective study carried out in the UK, with detection of viral RNA in two pools of mosquitoes, supporting the spread of the virus to new areas [10].

Effective control of MBDs requires a comprehensive understanding of pathogen transmission dynamics and epidemiology. Field investigations are indispensable for identifying the ecological components of transmission cycles, including reservoir hosts, vector species, and environmental conditions that sustain viral persistence. These studies provide critical insights into natural infection patterns and the influence of climatic and anthropogenic factors on vector populations [11]. However, complementary in vitro approaches, particularly experimental infections, can help to elucidate further interactions between viruses and their mosquito hosts. Hence, controlled laboratory infections facilitate the study of vector competence (VC) (proportions of vectors that become infected after exposure to an infected blood meal), viral replication kinetics, extrinsic incubation period (EIP)(the time that it takes the pathogen to develop inside the vector and become transmissible), dissemination pathways within the vector, and potential genetic adaptations that may enhance transmission efficiency [12,13]. Furthermore, these studies shed light on the physiological and behavioural impacts of infection on mosquitoes, offering valuable information for modelling vector competence and predicting outbreak risk. Therefore, by integrating field observations with experimental infection data, it is possible to develop robust, evidence-based strategies for surveillance and vector control, which ultimately will help to reduce the disease burden of emerging and re-emerging arboviruses.

In Europe, a number of vector competence studies have been performed in several *Aedes* and *Culex* mosquito species [13]. These studies are focused on the two main WNV lineages currently reported in Europe, WNV Lin1 and Lin2 [14]. More specifically, focusing on VC assessments for *Culex pipiens*, studies have predominantly tested one WNV lineage, and only Fros et al., 2015 [15], in the Netherlands, and Holler et al., 2026 [16], in Germany, have compared WNV Lin1 and Lin2. Some studies have investigated differences in the establishment of viruses and transmission in *Culex* mosquitoes maintained at different temperatures and doses [17,18,19,20,21]. In this study, we investigated colonised *Cx. pipiens* VC for WNV, conducting a series of controlled experimental infections to build on previous studies and assess and compare the influence of temperature (20 °C and 25 °C) on infection, dissemination and transmission of two WNV lineages. Mosquitoes were exposed to WNV strains representing Lin1 and Lin2 under varying temperature regimes and initial viral doses to assess the viral dynamics within this mosquito species, performing not only the detection of virus RNA, but also immunohistochemistry studies to detect the virus dissemination in the mosquito tissues. The temperatures assessed reflect average temperatures that are increasingly observed in the UK (and other temperate regions) during the summer months [22]. The data generated in this study will contribute to risk models aimed at improving our understanding and preparedness on the event of a WNV outbreak in northern European regions. Moreover, vectorial capacity, which describes the potential for a disease to be transmitted by a vector, depends on multiple factors, including VC and EIP. Both parameters are assessed in this study and will provide a more comprehensive understanding of the vectorial capacity of *Cx. pipiens* for WNV Lin1 and 2.

## 2. Materials and Methods

### 2.1. Mosquito Rearing

This research utilised a colony of *Cx. pipiens* s.l. (UK origin) established in 2011 and subsequently enriched with wild-caught specimens in 2022. The mosquitoes were reared in an insectary under controlled conditions, with temperature maintained at 25 °C, approximately 60% relative humidity, and a 12:12 h light–dark cycle. Adult mosquitoes were provided with a 10% sucrose solution ad libitum, and female mosquitoes received a weekly blood meal consisting of defibrinated horse blood (TCS Biosciences Ltd., Buckingham, UK) using a Hemotek device (Hemotek Ltd., Blackburn, UK). First instar (L1) larvae were initially fed on Liquifry (Interpet, Dorking, UK) and subsequently transitioned to fish food pellets (ExtraSelect, Su-Bridge Pet Supplies Ltd., Thetford, UK).

### 2.2. Virus Stocks

In this study, the virus strains assessed were WNV Lin1 strain Gsl98, isolated from a goose (*Anser anser*), and WNV Lin2 291.B/2013/Velky Biel/SVK, isolated from a goshawk (*Accipiter gentilis*) in Slovakia. Briefly, virus strains were propagated in Vero C1008 cells using culture medium consisting of Eagles minimal essential medium (E-MEM, Sigma Aldrich, Gillingham, UK), with 10% foetal bovine serum (FBS) and penicillin–streptomycin–nystatin solution (1%, Thermo Fisher Scientific, Horsham, UK) at 37 °C and 5% CO_2_ for 5–7 days. Infection of the cell monolayer was confirmed by light microscopy to assess the cytopathic effect (CPE). Virus titration was performed as explained below in Section 2.6.

### 2.3. Experimental Infections and Sample Collection

Female mosquitoes aged 5–10 days were deprived of sucrose solution during the morning of the experiment and exposed to an infected blood meal using a Hemotek device in the afternoon, allowing them to blood feed overnight. The infected blood meal (2 mL total volume) consisted of 1200 µL of defibrinated horse blood (TCS Biosciences Ltd., Buckingham, UK), 100 µL of dATP (Sigma-Aldrich, Gillingham, UK) at a concentration 20 µM, and 700 µL of WNV tissue culture supernatant (virus concentration adjusted to reach the required PFU/mL in the final volume). Experimental conditions and viral doses for each lineage are shown in Table 1. The following morning, the engorged females were separated from unfed mosquitoes, grouped into experimental cages and placed into incubators at the corresponding experimental temperature. One control cage (no infectious blood meal) was also included at each temperature. Mosquitoes were housed in the respective incubators and monitored regularly. Any deceased specimens were removed, recorded, and frozen as necessary, while sucrose cotton, dampened in 10% sucrose solution, was replenished or replaced as needed.

Mosquitoes were sampled at different time points: 7 days post-infection (DPI), 14 DPI, and/or 21 DPI. At each timepoint, mosquitoes were immobilised using FlyNap (Carolina Biological Supply Company, Burlington, NC, USA) and dissected individually to collect wings and legs, bodies (without head), and saliva in separate tubes containing E-MEM media supplemented with 10% FBS and a 1% penicillin–streptomycin–nystatin solution. After dissecting wings and legs, each mosquito was allowed to salivate by placing the proboscis into the end of a pipette tip containing 20 µL of tissue culture media consisting of E-MEM, 50% sucrose and dATP (20 µM). After ~30 min, the head was separated from the body and the body placed in a tube with supplemented E-MEM as described above. These samples were collected for the subsequent calculation of infection, dissemination and transmission potential rates, which are defined as the proportion of mosquitoes with infected midgut among mosquitoes exposed to the infectious blood meal, proportion of mosquitoes with disseminated viral particles (or viral RNA) outside the midgut and the proportion of mosquitoes showing viral particles (or viral RNA) in their saliva, respectively. Gathering these parameters, and more specifically the transmission potential, allows the investigation of the time that it takes for virus particles to be potentially transmitted through the bite after being ingested during an infective blood meal. This period from virus ingestion until transmission is known as the EIP.

Additionally, for each DPI and temperature, 2–5 mosquitoes were randomly selected for further immunohistochemistry (IHC) studies. In those samples, wings and legs were collected, the mosquitoes were allowed to salivate and then the body (with its head intact) placed in 10% neutral buffered formalin for a minimum of 10 days until further processing (see section below).

### 2.4. Immunohistochemistry

Formalin-fixed paraffin-embedded tissue blocks were serial sectioned at 3 μm thickness and stained with Haematoxylin and Eosin (H&E) and for WNV immunohistochemical (IHC) labelling. Slides were dewaxed and rehydrated through xylene and absolute alcohol, respectively, and quenched for endogenous peroxidase with 3% hydrogen peroxide in methanol (VWR International, Lutterworth, UK) for 15 min at room temperature (RT), before epitope unmasking using Target Retrieval Solution pH 9 buffer (Agilent Dako, Santa Clara, CA, USA) for 10 min at 100 °C by microwave (Thermo Shandon Ltd., Cheshire, UK). Slides were blocked with normal goat serum for 20 min at RT (1/66 dilution; Vector Laboratories, Redcar, UK) and assembled into cover plates to facilitate IHC using the Shandon Sequenza system (Thermo Shandon Ltd., Cheshire, UK). Samples were then incubated with Anti-Dengue Pan-Serotype NS1 antibody (Native Antigen, Kidlington, UK) at 0.17 µg/mL for 18 h at 4°c with concentration-matched isotype controls (Mouse IgG; Vector Laboratories), followed by incubation with mouse-specific Envision HRP labelled polymer (Agilent Dako, Santa Clara, USA) with an additional normal goat serum (1/66 dilution; Vector Laboratories) for 30 min at RT. According to the manufacturer, this Anti-Dengue Pan-Serotype NS1 antibody show cross-reaction with a broad range of orthoflaviviruses, such as WNV, and has previously been used by the authors to detect another orthoflavivirus, Usutu virus [23]. Immunolabelling was visualised using 3,3-diaminobenzidine tetrahydrochloride (DAB) (Sigma Aldrich, Gillingham, UK) for 10 min at RT. Tris-buffered saline (0.85% NaCl) with Tween (Fisher Scientific, VWR International, Lutterworth, UK) was used for rinsing sections between incubations and as the antibody diluent. Subsequently, sections were counterstained with Mayer’s haematoxylin (Surgipath, Leica Biosystems, Milton Keynes, UK), dehydrated and cleared in absolute alcohol and xylene, and glass coverslips mounted using Dibutyl Phthalate Xylene (TCS Biosciences Ltd., Buckingham, UK).

### 2.5. Sample Processing and Molecular Analysis

RNA extraction of all samples (legs/wings, bodies, and saliva) was carried out following a TRIzol^TM^ protocol (Thermo Fisher Scientific, Horsham, UK) [24]. Subsequently, viral RNA detection was performed by RT-qPCR [25] using an Agilent AriaMx Real-Time PCR System (Agilent, Santa Clara, USA). Briefly, the reaction mix includes: 2x iTaq Universal probes, WNV Fwd and Rv primer at 5 pmol/µL and WNV Probe at 2.5 pmol/µL, and iScript advance RT (BioRad, Watford, UK). Reaction conditions included a reverse transcription step at 50 °C for 10 min followed by an inactivation step at 95 °C for 5 min and subsequent PCR amplification step of 45 cycles 95 °C for 15sec and 55 °C for 30sec. Agilent Aria 1.8 software was used to analyse the results from the RT-qPCR.

### 2.6. Plaque Assays

Plaque assays for virus titration and for detection of viable virus in saliva samples were performed using Vero C1008 cells as previously described [26]. Briefly, 30 μL of each sample was used to set up the titration 10-fold dilutions. The dilutions were then transferred to 12-well plates containing Vero C1008 cells 95–100% confluency and incubated for 1 h at 37 °C, 5% CO_2_. After this time, a carboxy-methylcellulose (CMC) (Sigma-Aldrich, Gillingham, UK) overlay (0.5 mL 0.6% avicel in MEM) was applied and incubated for 6 days at 37 °C with 5% CO_2_. The overlay was supplemented with 100 μg/mL penicillin, 100 μg/mL streptomycin (Gibco, Thermo fisher Scientific, Horsham, UK), and L-glutamine 200 mM. After the incubation period, the cells were fixed and virus inactivated in 10% formaldehyde for 3 h and stained with 2.3% crystal violet. Plaques were counted in each well over a light box to determine the titre in plaque forming units per ml (PFU/mL).

### 2.7. Statistical Analysis

Statistical analyses were performed using the GraphPad Prism 8 software (GraphPad, San Diego, CA, USA). Fisher’s exact test was used to compare the infection, dissemination and transmission potential rates between both WNV lineages, as well as between the two temperatures tested for WNV Lin2. For WNV Lin1, where three temperatures were evaluated, differences between these rates were analysed using Chi-square test.

## 3. Results

### 3.1. Experimental Infections

A total of 999 female *Cx. pipiens* were included in the experiments conducted in this study, of which 779 were exposed to WNV Lin1 or Lin2 at one of the three doses mentioned above. An additional 220 females were used as negative controls (non-virus exposure). Details of the feeding rates for each exposure are provided in Table 2A. In brief, the rate of feeding success varied from 63.5% to 98.5%.

Mosquitoes were monitored regularly to record mortality, and samples collections were performed at 7, 14, 21 DPI. The number of females dissected at each time point for each experimental infection is shown in Table 2B. Survival rates varied between experiments and between controls and experimental groups. These differences in survival are likely due to handling effects (details are provided in Supplementary Figure S1).

### 3.2. Infection, Dissemination, and Transmission Potential Rates by WNV RT-qPCR

**Figure 1:**
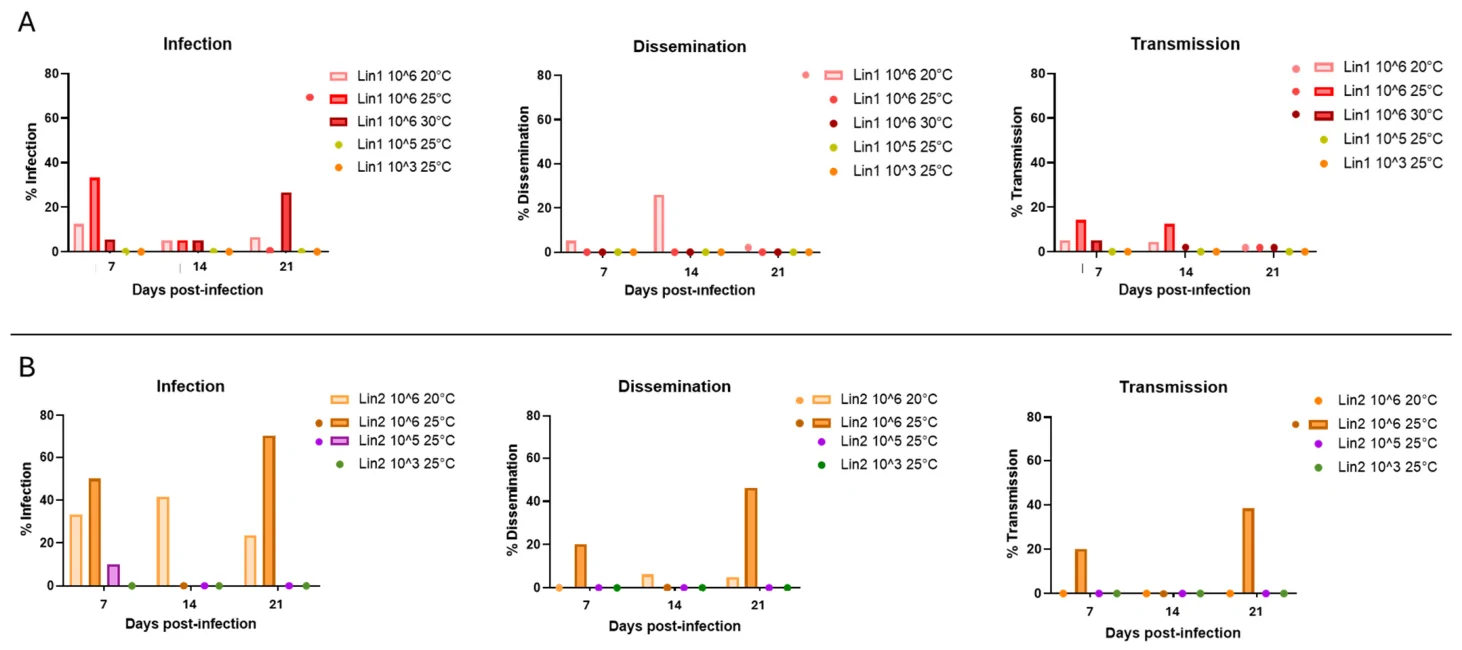
Graphs summarising infection, dissemination and transmission potential rates for mosquitoes exposed to (**A**) WNV Lin1 and (**B**) WNV Lin2.

Infection, dissemination and transmission potential were assessed as the proportion of mosquitoes’ bodies, wings/legs and saliva tested positive by WNV RT-qPCR (Figure 1; Supplementary Table S1).

In the VC assay using WNV Lin1 at a dose of 10^6^ PFU/mL, infection, dissemination, and transmission potential varied across temperatures and sampling points (Figure 1A). At 7 DPI, infection rates were highest at 25 °C (33.3%), compared with 12.5% at 20 °C and 5.5% at 30 °C. Dissemination was detected only in one mosquito at 20 °C. Transmission potential was detected across all three temperatures, with the highest rate again at 25 °C (14.3%). No significant differences were observed between temperatures at 7 DPI (Supplementary File S2). By 14 DPI, infection rates were similar across temperatures, ranging from 4.8% to 5%. Dissemination was identified only at 20 °C (26.1%), being highly significant when compared to the other two temperatures (*p*-value = 0.0009) (Supplementary File S2). Transmission potential at this point was highest at 25 °C (12.5%), compared with 4.3% at 20 °C, and undetectable at 30 °C. Finally, at 21 DPI, no dissemination or potential transmission was detected at any temperature. Infection was highest at 30 °C (26.6%), lower at 20 °C (6.6%), and absent at 25 °C (*p*-value = 0.0423) (Supplementary File S2).

In mosquitoes exposed to WNV Lin2 at a dose of 10^6^, the virus was detected at both temperatures tested (20 °C and 25 °C) (Figure 1B, Supplementary Table S1A). At 7 DPI, infection rate was higher at 25 °C (50%) compared to 20 °C (33.3%), while dissemination and transmission potential were only detected at 25 °C with a rate of 20% for both parameters. At 14 DPI, WNV detection tested positive in mosquitoes maintained at 20 °C with infection and dissemination rates of 41.6% and 6.3%, respectively. However, no saliva samples were positive by qRT-PCR. Notably, no viral RNA was detected in bodies, wings/legs, or saliva at 25 °C at this collection point. This absence may reflect random variation in sample collection or an issue during RNA extraction. Finally, at 21 DPI, WNV detection was consistently higher in samples from the 25 °C temperature treatment. Specifically, the infection rate at 25 °C was 60%, while at 20 °C it was significantly lower at 23.5% (*p*-value = 0.0402). Dissemination and transmission potential results at 25 °C showed 46.2% and 23.1% of samples positive to WNV, respectively, while there was no detection in samples at 20 °C. Dissemination and transmission potential at 25 °C were therefore highly significant, *p*-value = 0.0084 and 0.0064, respectively) (Supplementary File S2).

In the third experiment, which assessed viral doses of 10^3^ and 10^5^ PFU/mL for both WNV lineages at 25 °C, only one mosquito body was found to be positive by qRT-PCR. This mosquito belonged to the group exposed to WNV Lin2 at 10^5^ PFU/mL, corresponding to an infection rate of 10% (Figure 1, Supplementary Table S1B).

In summary, the viral dynamics of WNV Lin1 in temperate *Cx. pipiens* appeared relatively low, even at high doses, and was influenced by temperature. Transmission potential was only detected up to 14 DPI, with the highest rates at 25 °C, although transmission at 20 °C— temperatures commonly reached during northern European summers—was still possible.

Regarding WNV Lin2, the experiments performed in our study demonstrate greater infection, dissemination and transmission potential in temperate mosquitoes compared to WNV Lin1, showing highly significant differences between the two lineages at 21 DPI (*p*-value = 0.0001, 0.0015 and 0.0054 for infection, dissemination and transmission potential, respectively) (Supplementary File S2). Notably, virus was detected in a mosquito exposed to a dose as low as 10^5^ PFU/mL, suggesting the possibility of infection through exposure to lower titres of virus. Importantly, WNV Lin2 was detected by qRT-PCR in saliva at 25 °C at both 7 and 21 DPI, indicating sustained transmission potential. At 20 °C, infection rates remained high, but dissemination was reduced and no transmission potential was observed, suggesting that vector competence for WNV Lin2 in *Cx. pipiens* is enhanced at higher temperatures.

### 3.3. Detection of Viable Virus in Saliva by Plaque Assay

Saliva samples positive by WNV RT-qPCR were further assessed by plaque assay in order to detect viable virus. Saliva samples were only found positive in experiment 1 and experiment 2. From those, the samples within experiment 1 did not yield any detectable plaques as evidence of viable virus. However, for experiment 2, seven saliva homogenates were assessed by plaque assay. Four out of the seven samples yielded viral plaques, corresponding to one saliva from 7 DPI and three from 21 DPI. The higher titre (1.4 × 10^4^ PFU/mL) was found in the saliva homogenate from 7 DPI, which showcases the more rapid infection establishment and subsequent dissemination to the salivary glands of WNV Lin2. The virus titre range in samples from 21 DPI were lower: 3.7 × 10^1^, 1.6 × 10^3^ and 3 × 10^2^ PFU/mL. This result highlights the higher transmission potential of *Cx. pipiens* for WNV Lin2 (Table 3).

### 3.4. Detection of WNV by Immunohistochemistry

**Figure 2:**
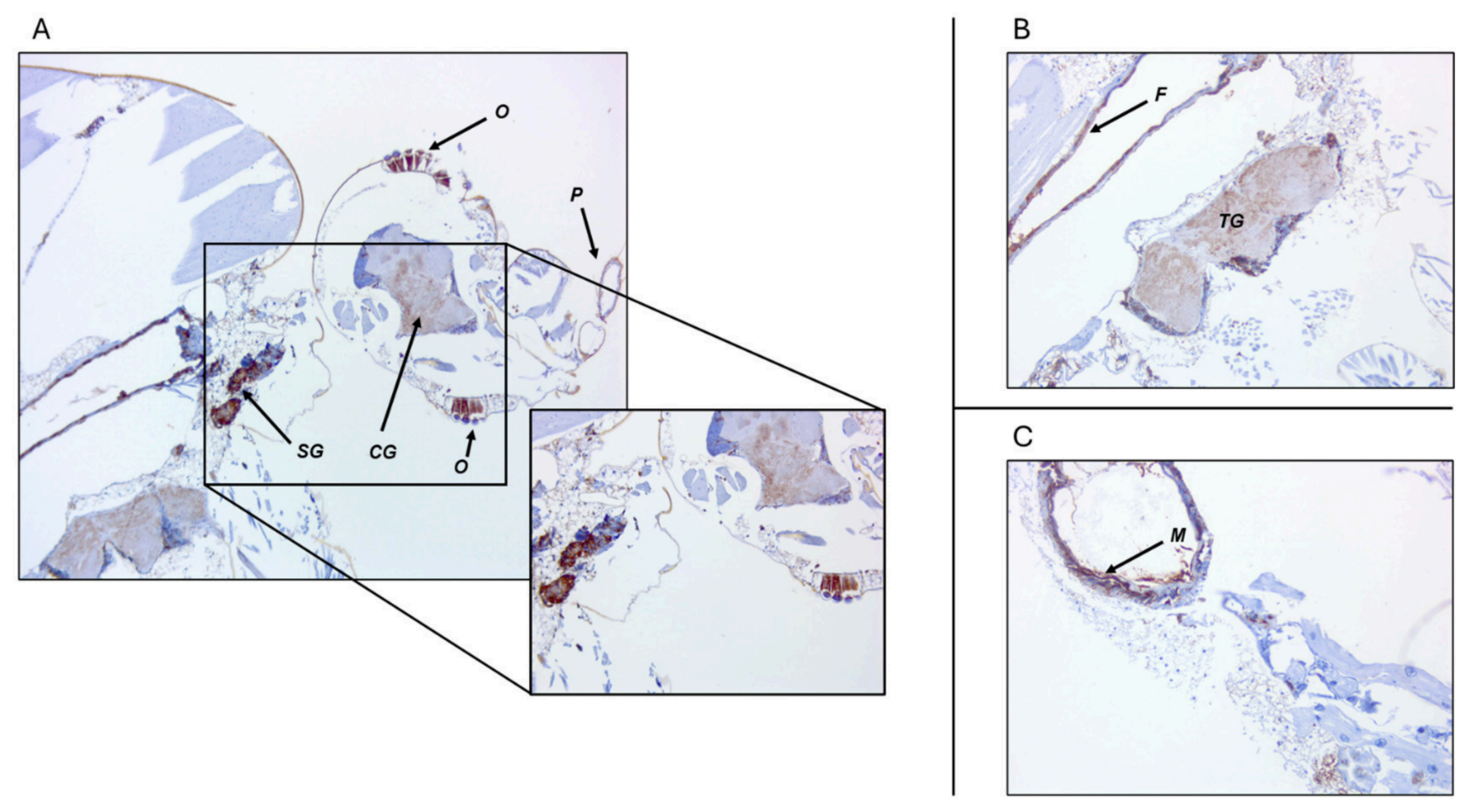
Immunohistochemistry for orthoflavivirus NS1 in the head and thorax of mosquito samples collected from experiment 2 (WNV lineage 2, 10^6^ PFU/mL, at 25 °C, 21 DPI). Viral antigen was identified by brown staining in (**A**) the ommatidia (O), salivary gland (SG), cerebral ganglia (CG), palps (P), (**B**) foregut (F), thoracic ganglia (TG) and (**C**) midgut (M).

A total of 29 mosquito bodies were submitted for IHC from experiment 1, 16 bodies for experiment 2, and 22 for experiment 3. From all the samples, detection was possible only in one of the stained mosquito bodies from experiment 2, with virus dissemination detected throughout several tissues. This sample corresponded to a Lin2-infected mosquito dissected at 21 DPI from the 25 °C temperature assessment, in which wings and legs, and saliva were also positive for WNV RT-qPCR confirming the infection, dissemination and transmission potential of the virus. In Figure 2, the staining indicates the dissemination of WNV antigen to different tissues of the infected mosquito, including the midgut, foregut, salivary glands, cerebral ganglia and ommatidia.

## 4. Discussion and Conclusions

From its introduction in Europe in mid-20th century, WNV has spread throughout the continent, affecting mostly countries of the Mediterranean region, but more recently being detected in temperate countries such as Germany, Belgium and the Netherlands, where cases in humans, horses and/or birds have been reported [5,8,27,28]. It has also been detected in mosquitoes in the UK [10]. In this study, we provide experimental evidence that *Cx. pipiens* mosquitoes from a long-established UK colony are competent vectors for both WNV Lin1 and Lin2 at temperatures currently recorded during northern European summers (20–25 °C) and projected future mean temperatures (30 °C). These findings reinforce existing concerns that the gradual warming of northern latitudes may create ecological conditions increasingly favourable for MBD transmission, including WNV, in regions that have historically remained free of disease circulation [29,30].

The results from our VC assays showed that across both viral lineages, 25 °C emerged as the most permissive temperature for infection establishment, viral dissemination and final transmission. This result agrees with previous studies demonstrating that VC in *Cx. pipiens* from temperate regions positively correlates with temperature and that temperatures below 20 °C typically reduce or significantly delay the EIP [13,18]. However, we observed differences between WNV lineages. WNV Lin2 displayed markedly higher infection, dissemination and transmission rates at 25 °C compared with Lin1, potentially reflecting its epidemiological success and geographical expansion throughout central and northern Europe in the past decade [14]. Conversely, Lin1, by contrast, produced lower overall rates but remained detectable at all three tested temperatures, suggesting that it may maintain a wider thermal tolerance, albeit at reduced efficiency. The experiments conducted at 30 °C, which were performed exclusively with WNV Lin1 as described in Section 2, resulted in high infection rates but no successful viral dissemination or transmission after 14 DPI. These findings contrast with those reported in several European studies, which observed transmission efficiencies at temperatures over 27 °C [16,20,31]. However, direct comparison is challenging because those studies used field-collected *Cx. pipiens* mosquitoes and maintained them under fluctuating daily temperature regimes; whereas, our experiments were conducted with colonised mosquitoes under constant laboratory conditions, which may potentially affect the mosquito response to the virus infection.

The contrast between the lineages aligns with the broader epidemiological pattern in Europe: WNV Lin2 has become the dominant lineage across much of the continent [14] and is associated with more frequent and geographically extensive outbreaks. There have been limited studies assessing WNV pathogenicity in birds, with research indicating similar effects of both lineages in birds of prey [32], while in partridges and house sparrows, Lin1 was found to be more pathogenic [33,34]. Hence, there is still insufficient evidence regarding lineage-specific differences in mosquito–virus interactions. However, our results suggest that even if Lin1 retains high pathogenicity for some avian species, Lin2 may have a competitive advantage within the mosquito vector, potentially contributing to its continued expansion.

Temperature-dependent differences in transmission dynamics further highlight the ecological implications of these findings. Results from the experiments with WNV Lin1 showed lower rates of infection and dissemination, yet the virus was still detectable, although at very low rates, in the saliva at 20 °C, suggesting potential transmission at cooler temperatures if the EIP is sufficiently long. Hence, for WNV Lin1, this *Cx. pipiens* population showed a limited capability of sustaining transmission at 20 °C. On the other hand, only WNV Lin2 produced detectable transmission at 25 °C in the mosquito population studied, but at substantially higher rates. This temperature threshold is consistent with previous studies reporting that Lin2 dissemination and EIP acceleration occur most efficiently above 23 °C [15,19]. In regions of Europe where summer temperatures often exceed 23 °C—even intermittently—Lin2 is therefore likely to achieve more efficient transmission, whereas Lin1 may retain a low but persistent transmission potential in cooler climates. This could have relevance for northern Europe, where projected temperature increases under climate change scenarios are expected to significantly elevate the risk of WNV circulation [29,30].

The findings in this study also highlighted the effect of infectious dose on VC, which is related to the level of viremia in the avian hosts. In this sense, WNV Lin1 required doses ≥ 10^6^ PFU/mL to establish infection reliably, consistent with previous studies showing that low infectious doses significantly reduce successful midgut infection and dissemination through the mosquito body [21,35]. In contrast, WNV Lin2 led to infection at 10^5^ PFU/mL, but no subsequent dissemination to legs and wings, or potential transmission. This result matched recent studies performed in *Cx. pipiens* biotype *pipiens* and *Cx. torrentium* mosquitoes suggesting that lower virus doses may be insufficient to sustain mosquito-mediated transmission cycles, particularly for Lin1, and that Lin2 may have a slightly lower minimum infection threshold but still requires high viral titre in blood meals for successful dissemination [16]. In this sense, studies where viral load was assessed in female mosquitoes collected after blood feeding, a 1–2 log decrease was detected [21,36]. However, the samples were frozen before processing, and the freeze–thaw process can lead to a reduction in viral titre. This factor, highlighted in our study, is one of the limitations of vector competence assays. Also, in natural ecosystems, where viral loads in wild birds fluctuate, these thresholds could influence the relative likelihood of local amplification for each lineage. Vaughan et al., 2022, investigated these fluctuations by comparing viral loads in mosquitoes exposed to experimentally infected birds inoculated with WNV NY99, with results that supported the importance of the avian host and the initial viral dose [37].

Immunohistochemistry supported the virological results by demonstrating widespread dissemination of WNV Lin2 virus throughout mosquito tissues, including the ommatidia and cerebral ganglia. More importantly, virus antigen was detected in the salivary glands, confirming the transmission potential findings through WNV RT-qPCR. Moreover, viral presence in neural tissues and sensorial organs have previously been associated with potential behavioural modification in mosquitoes, such as altered host-seeking or feeding persistence, although this remains understudied for WNV [38,39]. The low detection of WNV by IHC in the mosquitoes of this study could be related to the fact that the antibody used was not specific to WNV. Additionally, the plaque assay results confirmed the presence of infectious particles of WNV Lin2 in the saliva as early as 7 DPI, which aligns with previous observations that under optimal temperatures, the EIP of WNV in *Cx. pipiens* may be as short as 5–7 days [15,18]. The negative results observed in some saliva samples may be attributed to low initial viral titres, as well as the freeze–thaw process, which can compromise viral viability. For further verification of the RNA integrity of the samples, amplification of an endogenous mosquito gene and WNV RT-PCR was repeated on those mosquito samples with discrepancies, confirming the sample integrity and initial viral detection. Therefore, although our findings support the evidence of viral transmission, they only indicate the potential for transmission. Further optimisation of experimental protocols is required to better assess the infectivity of viral particles present in collected mosquito saliva.

The implications of these findings for the potential emergence of WNV in the UK are particularly noteworthy. Although the UK has not yet experienced sustained local transmission, the combination of an apparently competent *Cx. pipiens* population, increasingly favourable summer temperatures, and continued expansion of WNV Lin2 across mainland Europe suggests that conditions are gradually aligning for possible virus introduction and establishment. Modelling studies predict that northern Europe, including regions climatically similar to the UK, will experience substantial increases in ecological suitability for WNV circulation as mean summer temperatures rise and heatwave frequency intensifies [29,30]. The demonstration that UK *Cx. pipiens* can potentially support WNV infection and transmission at temperatures routinely recorded during recent British summers highlights the narrowing barrier preventing local emergence. Moreover, the continued westward spread of Lin2 from central Europe—driven in part by rapid bird migration routes and favourable land-use patterns [14]—means that eventual introduction of infectious birds or infected mosquitoes into the UK is increasingly plausible. These findings therefore reinforce the importance of active entomological and avian surveillance, early-season monitoring of mosquito infection dynamics, and preparedness for detection and response to potential WNV incursions. In this regard, vector surveillance programmes have increased, monitoring the mosquito population presence and expansion, and investigating bird and mosquito exposure to MBDs, such as WNV and USUV, with recent findings showing the wider distribution of the proven bridge vector *Cx. modestus* and the detection of WNV RNA in UK *Ae. vexans* through retrospective testing [10,40,41].

Overall, our findings add to the growing body of evidence that *Cx. pipiens* populations in northern Europe possess the capacity to transmit both major WNV lineages under current summer temperatures. Nonetheless, more investigation is needed since the VC results from this study are only one of the factors involved in the bigger picture of vectorial capacity that includes other parameters such as host and vector densities, survival rates or biting rates. Hence, further investigation and continued monitoring of mosquito vectors, avian host infection dynamics, presence of other potential hosts alongside long-term climate projections, will be essential for anticipating and mitigating WNV introduction and establishment in new regions.

## Figures and Tables

**Table 1:** Experimental conditions and doses of West Nile virus lineages 1 and 2 used in each of the experiments. (DPI: days post-infection). Due to time and logistic impediments, it was not possible to carry out the VC assay for WNV Lin2 at 30 °C.

Experiment	Lineage	Dose (PFU/mL)	Temperature (°C)	Sample Collection Points (at DPI)
1	Lineage 1 strain Gsl98	2.59 × 10^6^	20, 25, 30	7, 14, 21
2	Lineage 2 291.B/2013/Velky Biel/SVK	3.85 × 10^6^	20, 25	7, 14, 21
3	Lineage 1 strain Gsl98	2.6 × 10^5^	25	7, 14
		2.6 × 10^3^		
	Lineage 2 291.B/2013/Velky Biel/SVK	3.85 × 10^5^		
		7.7 × 10^3^		

**Table 2:** Tables showing a breakdown of mosquito numbers for each assay. (A) Number of female mosquitoes exposed to uninfected and infected blood meals and the resultant total of engorged females (blood feeding rates, %) for each of the experiments. (B) Number of mosquitoes sampled in each experiment at each timepoint (days post-infection, DPI).

(A)					
Experiment	Treatment	Total Mosquitoes Exposed		Total Blood Fed (%)	
1	Control	85		54 (63.5)	
	Lin1 10^6^	310		206 (66.5)	
2	Control	88		59 (67.1)	
	Lin2 10^6^	207		176 (85.0)	
3	Control	47		44 (93.6)	
	Lin1 10^5^	74		72 (97.3)	
	Lin1 10^3^	68		67 (98.5)	
	Lin2 10^5^	59		50 (84.7)	
	Lin2 10^3^	61		57 (93.4)	
**(B)**					
**Experiment**	**Treatment**	**Temperature (°C)**	**DPI7**	**DPI 14**	**DPI 21**
1	Control	20	-	-	11
		25	-	-	8
		30	-	-	8
	Lin1 10^6^	20	19	23	18
		25	21	24	20
		30	20	25	18
2	Control	20	3	3	10
		25	3	3	3
	Lin2 10^6^	20	15	16	20
		25	12	10	13
3	Control	25	-	10	-
	Lin1 10^5^	25	15	20	-
	Lin1 10^3^	25	15	20	-
	Lin2 10^5^	25	12	20	-
	Lin2 10^3^	25	12	8	-

**Table 3:** Results from the plaque assay titration of the saliva samples positive to WNV RT-qPCR. (DPI, days post-infection; PFU, plaque forming units per mL).

Assay	Sample	DPI	Temperature (°C)	Virus Titre (PFU/mL)
**WNV Lin1 10^6^**	Saliva 15	7	20	0
	Saliva 4	7	25	0
	Saliva 6	7	25	0
	Saliva 20	7	25	0
	Saliva 7	14	20	0
	Saliva 5	14	25	0
	Saliva 13	14	25	0
	Saliva 20	14	25	0
**WNV Lin2 10^6^**	Saliva 6	7	25	3.5 × 10^4^
	Saliva 7	7	25	0
	Saliva 2	21	25	3.7 × 10^1^
	Saliva 9	21	25	1.6 × 10^3^
	Saliva 10	21	25	0
	Saliva 11	21	25	0
	Saliva 13	21	25	3.0 × 10^2^

## Data Availability

All data generated is available in the manuscript figures, tables and Supplemental Files.

## Supplementary Materials

The following supporting information can be downloaded at: https://www.mdpi.com/article/10.3390/microorganisms14092084/s1.

## Abbreviations

The following abbreviations are used in this manuscript:

DAB	3,3-diaminobenzidine tetrahydrochorlide
DPI	Days post-infection
EIP	Extrinsic Incubation Period
EMEM	Eagle’s Minimum Essential Medium
H&E	Haematoxylin and Eosin
IHC	Immunohistochemistry
Lineage 1	Lin1
Lineage 2	Lin2
MBD	Mosquito-borne disease
PFU	Plaque-formation unit
RT	Room temperature
UK	United Kingdom
USUV	Usutu virus
VC	Vector competence
WNV	West Nile virus
