# Supplementary material for: Vector Competence for West Nile Virus Lineage 1 and 2: Temperature Dependency in Temperate Culex pipiens Mosquitoes
Source: Microorganisms. 2026 Sep 17;14(9):2084. doi: 10.3390/microorganisms14092084 (PMC13609240; doi:10.3390/microorganisms14092084)
Supplement: Supplementary file 1 [file microorganisms-14-02084-s001.zip › Supplementary File S1.pdf]

Vector competence for West Nile virus lineage 1 and 2: temperature dependency in temperate *Culex pipiens*

Supplementary Files

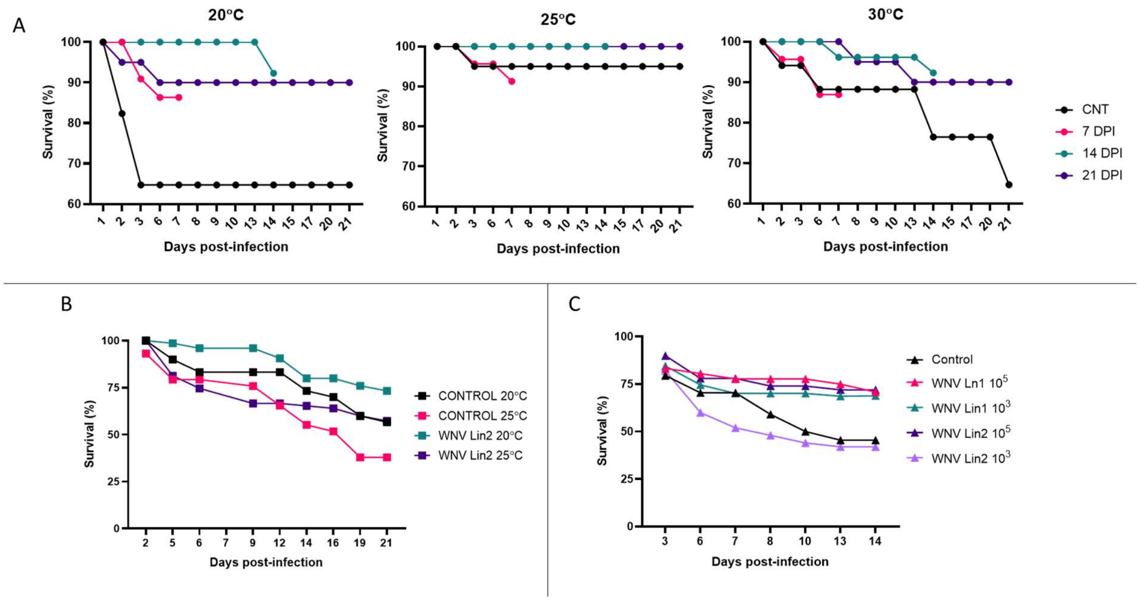

Supplementary Figure S1. Mosquito survival across the three experiments. **(A)** Experiment 1, experimental infection with WNV lineage 1, 10^6 PFU/ml, assessed at 20°C, 25°C and 30°C; **(B)** Experiment 2, experimental infection with WNV lineage 2, 10^6 PFU/ml, assessed at 20°C and 25°C; and **(C)** Experiment 3, experimental infection with both WNV lineages at doses of 10^5 and 10^3 PFU/ml, assessed at 25°C.

Supplementary Table S1. RT-qPCR results. **(A)** Results for experiments 1 and 2 showing rates of infection, dissemination and potential for transmission in *Culex pipiens* experimentally infected with WNV lineage 1 or lineage 2 dose of 10^6 PFU/ml. **(B)** Results for experiment 3 showing rates of infection, dissemination and potential for transmission in *Cx. pipiens* experimentally infected with WNV lineage 1 or lineage 2 doses of 10^3 and 10^5 PFU/ml. DPI (days post-infection)

A

		WNV lineage 1						WNV lineage 2					
		Infection		Dissemination		Transmission potential		Infection		Dissemination		Transmission potential	
DPI	Temp. (°C)	Body*		Legs and wings		Saliva		Body*		Legs and wings		Saliva	
		(n)	(%)	(n)	(%)	(n)	(%)	(n)	(%)	(n)	(%)	(n)	(%)
7	20	2	12.5	1	5.3	1	5.3	4	33.3	0	0	0	0
	25	6	33.3	0	0	3	14.3	5	50.0	2	20.0	2	20.0
	30	1	5.5	0	0	1	5.0	nd	nd	nd	nd	nd	nd
14	20	1	5.0	6	26.1	1	4.3	5	41.6	1	6.3	0	0
	25	1	4.8	0	0	3	12.5	0	0	0	0	0	0
	30	1	5.0	0	0	0	0	nd	nd	nd	nd	nd	nd
21	20	1	6.6	0	0	0	0	4	23.5	0	0	0	0
	25	0	0	0	0	0	0	6	60.0	6	46.2	3	23.1
	30	4	26.6	0	0	0	0	nd	nd	nd	nd	nd	nd

B

		**WNV lineage 1**						**WNV lineage 2**					
		Infection		Dissemination		Transmission potential		Infection		Dissemination		Transmission potential	
DPI	Dose	Body*		Legs and wings		Saliva		Body*		Legs and wings		Saliva	
		(n)	(%)	(n)	(%)	(n)	(%)	(n)	(%)	(n)	(%)	(n)	(%)
7	10^3	0	0	0	0	0	0	0	0	0	0	0	0
	10^5	0	0	0	0	0	0	1	10	0	0	0	0
14	10^3	0	0	0	0	0	0	0	0	0	0	0	0
	10^5	0	0	0	0	0	0	0	0	0	0	0	0

*Number of bodies analysed by RT-qPCR is less than the numbers of legs and wings and saliva assessed due to the sampling of bodies for immunohistochemistry. (See Supplementary Table2 and Table2B)

Supplementary Table S2. Number of mosquito bodies fixed in neutral buffered formalin for immunochemistry analysis in each experiment and at each timepoint (DPI: days post-infection).

Experiment	Treatment	Temperature (°C)	7 DPI	14 DPI	21 DPI
**1**	Control	20	-	-	3
		25	-	-	3
		30	-	-	3
	Lin1 10^6	20	3	3	3
		25	3	4	3
		30	2	5	3
**2**	Control	20	-	-	-
		25	-	-	-
	Lin2 10^6	20	3	3	3
		25	2	2	3
**3**	Control	25	-	-	-
	Lin1 10^5	25	3	3	-
	Lin1 10^3	25	3	3	-
	Lin2 10^5	25	2	3	-
	Lin2 10^3	25	2	3	-
